# Prevalence and determinants of hypertension control among almost 100 000 treated adults in the UK

**DOI:** 10.1136/openhrt-2020-001461

**Published:** 2021-03-11

**Authors:** Neo Tapela, Jennifer Collister, Lei Clifton, Iain Turnbull, Kazem Rahimi, David J Hunter

**Affiliations:** 1 Nuffield Department of Population Health, University of Oxford, Oxford, Oxfordshire, UK; 2 Botswana-Harvard AIDS Institute Partnership, Gaborone, Botswana; 3 Deep Medicine, Nuffield Department of Reproductive and Women’s Health, University of Oxford, Oxford, Oxfordshire, UK

**Keywords:** hypertension, outcome assessment, health care, epidemiology

## Abstract

**Objective:**

To identify factors associated with hypertension control among treated middle-aged UK adults.

**Methods:**

A cross-sectional population-based study including 99 468 previously diagnosed, treated hypertensives enrolled in the UK Biobank. Hypertension control was defined as systolic blood pressure <140 mm Hg and diastolic blood pressure <90 mm Hg.

**Results:**

Median age was 62.3 years (IQR 57.3 to 66.0), 45.9% female, 92.0% white, 40.1% obese, 9.3% current smokers and 19.4% had prior cardiovascular disease. 38.1% (95% CI 37.8% to 38.4%) were controlled. In multivariable logistic regression, associations with lack of hypertension control included: older age (OR 0.61, 95% CI 0.58 to 0.64 for 60–69 years compared with age 40–50 years), higher alcohol use (OR 0.61, 95% CI 0.58 to 0.64, for consuming >30 units per week compared with none), black ethnicity (OR 0.73, 95% CI 0.65 to 0.82 compared with white), obesity (OR 0.73, 95% CI 0.71 to 0.76 compared with normal body mass index). The strongest positive association with control was having ≥3 comorbidities (OR 2.09, 95% CI 1.95 to 2.23). Comorbidities associated with control included cardiovascular disease (OR 2.11, 95% CI 2.04 to 2.19), migraines (OR 1.68, 95% CI 1.56 to 1.81), diabetes (OR 1.32, 95% CI 1.27 to 1.36) and depression (OR 1.27, 95% CI 1.20 to 1.34).

**Conclusions:**

In one of the largest population-based analyses of middle-aged adults with measured blood pressure, the majority of treated hypertensives were uncontrolled. Risk factors for hypertension were associated with a lower probability of control. Having a comorbidity was associated with higher probability of control, possibly due to more frequent interaction with the healthcare system and/or appropriate management of those at greater cardiovascular risk.

**Key questions**

**What is already known about this subject?**

- Hypertension is prevalent in middle-aged adults in high-income countries. Factors influencing hypertension control in this population may differ from those in younger adults, and studies have been mixed on the association between comorbidities and hypertension control.

**What does this study add?**

- Our study is one of the largest population-based analyses of middle-aged adults treated for hypertension. Our findings suggest high levels of uncontrolled hypertension and identify characteristics associated with lower probability of hypertension control. Our findings may inform further investigations needed to better understand barriers to hypertension control, and contribute to the limited evidence on the association between comorbidities and hypertension control.

**How might this impact on clinical practice?**

- Our findings may help to identify subgroups for which clinical practice improvement efforts can be targeted.

## Introduction

Hypertension is the leading preventable risk factor for cardiovascular disease (CVD) mortality, affecting over 1.3 billion people around the world^1^ and responsible for approximately half of all strokes and ischaemic cardiac events.^2^ Clinical trials have demonstrated that lowering blood pressure (BP) reduces the incidence of stroke by 35%–40%, myocardial infarction by 20%–25% and heart failure by 50%.^3^ Despite this, and the availability of low-cost treatments for hypertension,^4^ many hypertensives are undiagnosed or inadequately treated.^4–6^ More evidence on factors influencing hypertension control is needed to support the efforts of clinicians and policymakers to reduce the CVD burden.

Previous studies have reported on factors associated with hypertension control in the general adult population in the UK.^7^ Determinants of hypertension control may differ between younger and older adults.^8^ Better understanding of the factors associated with hypertension control is particularly needed for middle-aged and older adults, for whom hypertension is more prevalent but achieving control may be more challenging.^9^ Comorbidities and multimorbidity may be important factors influencing hypertension control in older people.^7^ Multimorbidity is defined as the concurrent presence of two or more long-term conditions,^10^ and is of increasing public health concern globally given its rising prevalence in the context of longer life expectancy and higher disease-specific survival rates. Multimorbidity has been associated with higher health service utilisation, social deprivation and increased mortality.^10^ However, multimorbidity is relatively understudied, and the existing studies have focused on the prevalence or clustering of multimorbidity or its impact on general health outcomes.^10–12^ The few studies investigating the relationship of multimorbidity with hypertension control have been inconsistent in their findings, particularly whether non-cardiovascular comorbidities are associated with better hypertension control.^7 13^

We aimed to: (1) determine the prevalence of hypertension control among UK adults aged 40–69 years old previously diagnosed with hypertension and currently on antihypertensive treatment and (2) identify factors associated with hypertension control (primary objective), including whether multimorbidity and specific comorbidities were associated with hypertension control.

## Methods

### Design and study population

We analysed baseline survey data from the UK Biobank (UKB), a large population-based prospective cohort study that recruited via mail 500 000 adults aged 40–69 years residing within 40 km of 22 assessment centres across England, Scotland and Wales between 2006 and 2010.^14^ Participation required presenting to the assessment centres and providing written informed consent. Participants who had completed the baseline survey, and reported previously being informed by a health professional that they had hypertension (aware), and reported use of antihypertensive medications (treated) were included in this analysis. We specified exclusion criteria a priori and excluded participants who were pregnant, had fewer than two BP measurements at the baseline visit or had implausible BP values (defined as previously reported^15^: systolic BP <70 mm Hg or ≥270 mm Hg, diastolic BP <50 mm Hg or ≥150 mm Hg). We additionally excluded participants who had a medical history notable for a condition: associated with poor prognosis (kidney failure, heart failure, liver failure, cancer other than skin); or for which the goals of care might take priority over hypertension control (eg, suicide attempt); or for which the participant may have required additional support from a caretaker for hypertension management, acknowledging that the UKB database did not include measures of severity of these conditions (schizophrenia, dementia, Parkinson’s disease, multiple sclerosis, myasthenia gravis, motor neuron disease, other demyelinating disease).

### Procedures and definitions

The UKB baseline information was gathered through (1) a self-administered computer touch screen structured questionnaire at survey centres, followed by (2) same-day in-person structured interview by a trained nurse, which was then followed by (3) physical measurements by a trained nurse (BP, weight, waist circumference). The in-person interview was coupled with review of the participant’s medication list, which participants had been asked to bring with them (over 80% of UKB participants complied). The survey collected information on sociodemographic characteristics, lifestyle health-related behaviour, medical history, family history of CVD and previous health screenings. BP measurement was performed twice, 1 min apart, with the participant in a sitting position and using an Omron HEM 7015-T automated sphygmomanometer. Participants with elevated BP (or other abnormal findings) were provided with a print-out of their results and advised to follow-up with their general practitioner.

Hypertension control was defined as having a mean systolic BP <140 mm Hg and diastolic BP <90 mm Hg, among individuals who reported previously being informed of a hypertension diagnosis by a health professional (aware) as well as use of antihypertensives (treated). The BP treatment target used is consistent with the UK National Institute for Health and Care Excellence (NICE) guidelines for hypertension management during the study period (NICE 2006) and other guidelines such as the United States’ seventh Report of the Joint National Committee on High BP, WHO-International Society of Hypertension and the European Society of Hypertension.^16^

Reported use of antihypertensives was via either one of two means. First, selection of ‘BP medication’ in response to the touchscreen question ‘Do you regularly take any of the following medications?’ Second, report during the interview of use of medications that are antihypertensives and which were subsequently assessed to be ‘probably for hypertension indication’ based on an antihypertensive treatment rubric we developed. This rubric was based on the NICE 2006 guidelines and employed the Anatomical Therapeutic Chemical classification system,^17^ which has been endorsed by the WHO and has been similarly applied in a previous UKB publication.^18^ In applying this rubric, we classified hypertensives as on antihypertensives ‘probably for hypertension indication’ if they were on medications in the first to fourth lines of treatment in 2006 NICE clinical algorithm, but did not report a diagnosis that was an alternate indication for the medications (eg, diabetes for ACE inhibitors).

Variables that were included in the analyses were sociodemographic characteristics, known or possible determinants of CVD (alcohol intake, smoking, physical activity, body mass index (BMI)) or hypertension control (number of comorbidities, types of comorbidities, number of antihypertensive medications, prior colorectal cancer screening as a proxy for healthcare utilisation). BMI was calculated by dividing weight by height squared (kg/m^2^) and categorised as: underweight <18.5 kg/m^2^, normal 18.5–24.9 kg/m^2^, overweight 25.0–29.9 kg/m^2^ and obese ≥30.0 kg/m^2^. Standard alcohol units (alcohol by volume equivalents) were derived from participant responses of the number of typical volume drinks for each type of alcohol consumed per week (eg, pint of beer, glass of wine, measure/shot of spirits/liquors). Physical activity was assessed using adapted questions from the validated short International Physical Activity Questionnaire^19^; the time spent in vigorous, moderate and walking activity was weighted by the energy expended for these categories of activity, to produce total metabolic equivalent task minutes per week. The Townsend deprivation index, based on the geographic unit of census output areas, is a measure of socioeconomic material deprivation that combines four variables routinely available in census data (unemployment, non-ownership of a car, non-ownership of a home and overcrowding at home) and strongly correlates with mortality.^20^ Education categories followed the scales used in the International Standard Classification of Education, while occupation categories followed the UK Office of National Statistics’ Standard Occupational Classification system.

In selecting comorbidities to be analysed, we took into consideration the prevalence of each condition in the middle-aged population of the UK, its clinical significance, as well as its inclusion in previous multimorbidity studies^10 12 21^ and the UK’s Quality Outcomes Framework—a pay-for-performance scheme to incentivise quality care by general practitioners. Conditions thus selected spanned cardiometabolic, respiratory, psychiatric and neurological systems. CVD was defined as ischaemic heart disease, stroke or transient ischaemic attack.

### Statistical analysis

Descriptive analyses were performed to compute the proportion of hypertension control, overall and stratified. Logistic regression models were fitted to compute unadjusted, age-adjusted and sex-adjusted and multiply-adjusted ORs and 95% CIs of explanatory variables. Sensitivity analysis was performed using only the second BP measurement (which tended to be lower than the first measurement). Exploratory analyses were performed to interrogate potential explanations for the results, the impact of excluding those with serious comorbidities, and effect modification by age group, number of comorbidities and presence of prior CVD. Agreement of BP measurements over time was assessed using Spearman correlation coefficients, for all UKB participants who had repeat BP measured within 3 years of the baseline visit (n 2134, or 0.4% of all UKB participants). All analyses were performed using R V.3.6.2.^22^

## Results

### Participant characteristics

**Figure 1:**
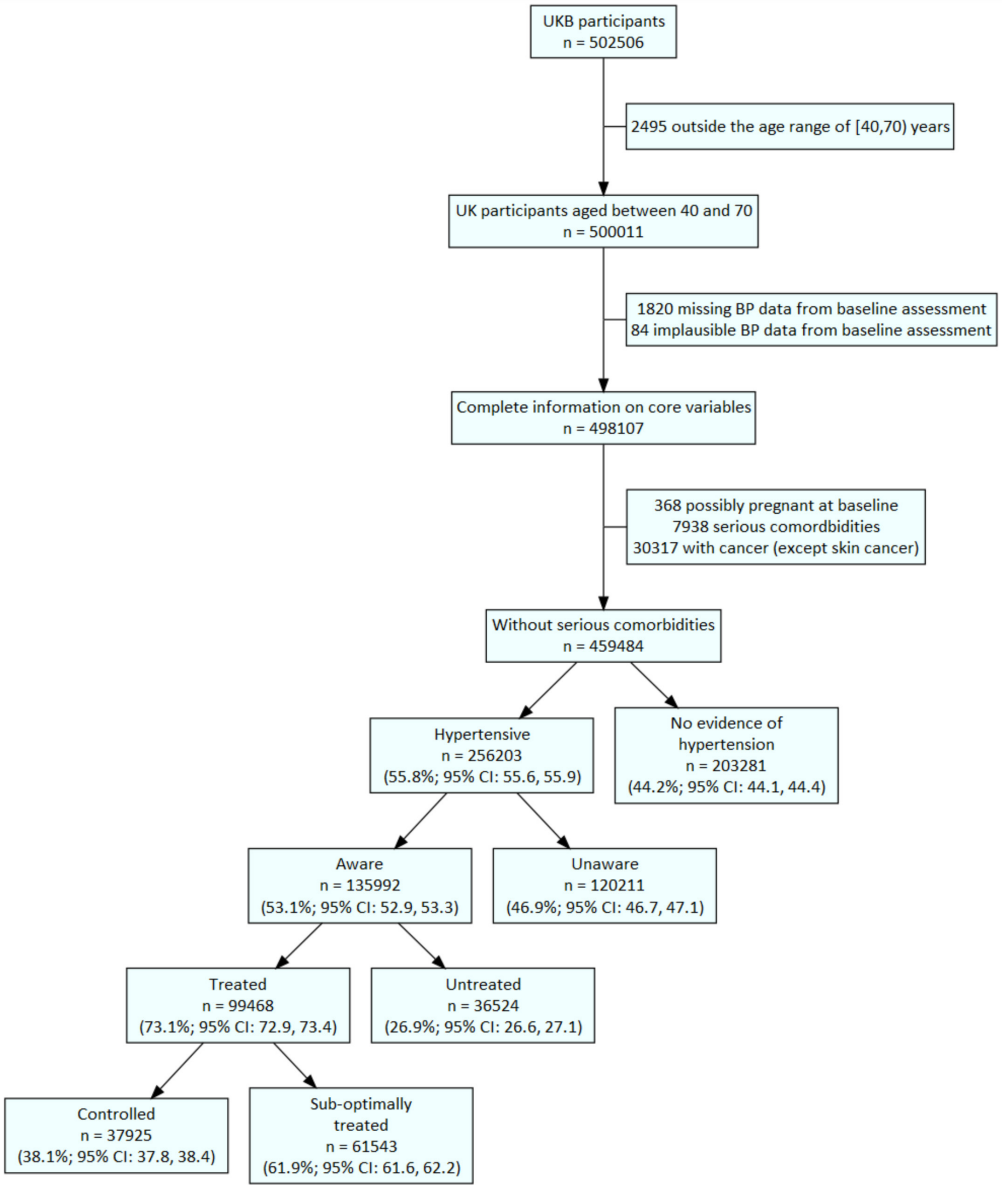
Flow chart illustrating selection of analytical dataset (n 99 468). Missing BP data comprises: having fewer than two BP measurements at baseline assessment or missing responses to questions on hypertension history. The following BP values were deemed implausible (defined as previously reported (15): systolic BP <70 mm Hg or ≥270 mm Hg, diastolic BP <50 mm Hg or ≥150 mm Hg. Hypertension was defined as self-report of hypertensive medication use, or self-report of a prior diagnosis of hypertension, or mean BP ≥140/90 mm Hg at baseline assessment. Awareness was defined as report of a prior diagnosis of hypertension by a health professional, among those who were hypertensive. Treatment was defined as report of hypertensive medication among those who were aware. Control was defined as mean BP <140/90 mm Hg at baseline assessment, among those who were treated. Hypertensives who were aware and treated but had BP ≥140/90 mm Hg were classified as inadequately treated. The BP diagnostic threshold and treatment targets used are consistent with the UK NICE guidelines for hypertension management during the study period (NICE 2006) and other guidelines such as the USA’s 7th Report of the Joint National Committee on High BP, WHO-International Society of Hypertension and the European Society of Hypertension.^16^ BP, blood pressure; NICE, National Institute for Health and Care Excellence; UKB, UK Biobank.

Out of 502 506 enrolled UKB participants, 99 468 were previously treated hypertensives who met inclusion and exclusion criteria for this analysis (figure 1). The median age in this group was 62.3 years (IQR 57.3–66.0 years), with 45.9% (45 607) of them female, 92.9% (92 385) white and 25.7% (25 606) having primary school as their highest attained education (table 1). A fifth (19.4%; 19 344) reported previous diagnosis of CVD, 40.1% (39 887) were obese and 9.3% (9254) were current smokers. The median duration of hypertension diagnosis was 7.3 years (IQR 3.6–12.6 years); 13.9% of all treated hypertensives were on ≥3 antihypertensives. Among the 19 344 treated hypertensives with CVD, 19.3% (3740) were on ≥3 antihypertensives; among the 79 022 treated hypertensives without CVD who were not smokers and were not obese (lower risk), 12.5% (9886) were on ≥3 antihypertensives (online supplemental table 1).

10.1136/openhrt-2020-001461.supp1 Supplementary data

**Table 1:** Characteristics of participants included in the analysis (treated hypertensives, N=99 468)

Variable	Levels (for categorical)	n	%
Median age, years (IQR)		62.3	(57.3–66.0)
Age group, years	40–49	7717	7.8
	50–59	27 823	28.0
	60–69	63 928	64.3
Gender	Female	45 607	45.9
	Male	53 861	54.1
Ethnic group	White	92 385	92.9
	Black	2362	2.4
	S.Asian	2118	2.1
	Mixed	458	0.5
	Other	1641	1.6
	Unanswered	504	0.5
Household income, GBP	Greater than 100 000	2529	2.5
	52 000 to 1 00 000	11 039	11.1
	31 000 to 51 999	18 368	18.5
	18 000 to 30 999	23 956	24.1
	Less than 18 000	26 443	26.6
	Do not know	5353	5.4
	Unanswered	11 780	11.8
Occupation category*	Professional and administrative	33 464	33.6
	Skilled trades	4232	4.3
	Services	5078	5.1
	Manual and industrial	6443	6.5
	Other employment	3118	3.1
	Retired	37 081	37.3
	Unable to work because of sickness or disability	5304	5.3
	Unemployed/unanswered	4748	4.8
Highest level of education (ISCED)†	5: Tertiary	38 924	39.1
	4: Post-secondary non-tertiary	12 224	12.3
	2–3: Secondary	20 635	20.7
	1: Primary	25 606	25.7
	Unanswered	2079	2.1
UK country of residence‡	England	88 122	88.6
	Scotland	7109	7.1
	Wales	4237	4.3
BMI (categorical)§	Underweight	197	0.2
	Normal (ref)	17 206	17.3
	Overweight	41 574	41.8
	Obese	39 887	40.1
	Unanswered	604	0.6
Smoking status	Never	48 974	49.2
	Previous	40 595	40.8
	Current	9254	9.3
	Unanswered	645	0.6
Median reported duration of hypertension, years (IQR)		7.3	(3.6–12.6)
No of antihypertensive medications	1	48 501	48.8
	2	33 678	33.9
	≥3	13 836	13.9
	Medication list unavailable	3453	3.5
Cardiovascular disease	No	80 124	80.6
	Yes	19 344	19.4
Diabetes	No	84 040	84.5
	Yes	15 428	15.5
No of comorbidities¶	0	44 968	45.2
	1	36 901	37.1
	2	13 583	13.7
	≥3	4016	4.0

*Occupation categories have been condensed from those recorded in UKB. Professional and administrative: managers and senior officials, professional occupations, associate professional and technical occupations, administrative and secretarial occupations. Skilled trades: skilled trades occupations. Services: personal service occupations, sales and customer service occupations. Manual and industrial: process, pPlant and machine operatives, elementary occupations. Other employment: free text entry that was not coded by UKB.

†Self-reported highest education achieved was mapped to the ISCED categories.

‡The assessment centre the participant attended was used as a proxy for country of residence.

§BMI has been categorised as: underweight <18.5 kg/m^2^; normal 18.5–24.9 kg/m^2^; overweight 25.0–29.9 kg/m^2^; obese ≥30.0 kg/m^2^.

¶The conditions that counted towards the number of comorbidities were: cardiovascular disease, diabetes, arrhythmia (afib/flutter), asthma or COPD, migraines, epilepsy, anxiety, depression, osteoarthritis, other joint disorder.

BMI, body mass index; COPD, chronic obstructive pulmonary disease; ISCED, International Standard Classification of Education; UKB, UK Biobank.

### Prevalence of hypertension control

Among all individuals aged 40–69 year including in this analysis (459, 484), we found a hypertension prevalence of 55.8% (95% CI 55.6% to 55.9%; 256 203), as well as notable gaps in the hypertension care cascade (figure 1). Nearly half (46.9%, 95% CI 46.7% to 47.1%; 120 211) of hypertensives were unaware of their condition; 26.9% (95% CI 26.6% to 27.1%; 36 524) of those who were aware were untreated; only 38.1% (95% CI 37.8% to 38.4%; 37 925) of treated hypertensives were controlled (61 543 or 61.9% were suboptimally treated). When employing a more conservative threshold of BP <160/100 mm Hg to define hypertension control, we found that 20 573 or 20.7% (95% CI 20.4% to 20.9%) were suboptimally treated (online supplemental figure 1). In fact, 3.8% (3754) of treated hypertensives had a systolic BP ≥180 or diastolic BP ≥110 mm Hg (online supplemental figure 2).

10.1136/openhrt-2020-001461.supp2 Supplementary data

10.1136/openhrt-2020-001461.supp3 Supplementary data

Regression to the mean, random within-person variation, white coat hypertension and changes in lifestyle or medications notwithstanding, our analysis indicates good concordance over time (Spearman’s rank correlations of 0.72 for systolic BP and 0.70 for diastolic BP for repeat measurements within 3 years) and high probability (73.1% agreement) that those classified as hypertensive at baseline using the 140/90 mm Hg threshold would have been classified similarly with a follow-up BP measurement (online supplemental table 2).

### Factors associated with hypertension control

Hypertension control was inversely associated with known risk factors of hypertension: older age (multiply-adjusted OR 0.61, 95% CI 0.58 to 0.64 for age 60–69 years compared with reference age group 40–49 year olds), male gender (OR 0.92, 95% CI 0.89 to 0.95), higher alcohol use (OR 0.61, 95% CI 0.58 to 0.64 for consumption of over 30 units per week), black ethnicity (OR 0.73, 95% CI 0.65 to 0.82 compared with White ethnicity), and obesity (OR 0.73, 95% CI 0.71 to 0.76) (table 2).

**Table 2:** Multivariable logistic regression identifying factors associated with hypertension control, among middle-aged UK adults on antihypertensive treatment (N=99 468)

				Age and sex adjusted			Multivariable adjusted		
Coefficient	Level	n*	% controlled†	OR	95% CI	P value	OR	95% CI	P value
Age group, years	40–49	7717	46.4	1			1		
	50–59	27 823	42.1	0.84	(0.80 to 0.88)	<0.001	0.82	(0.78 to 0.86)	<0.001
	60–69	63 928	35.4	0.64	(0.61 to 0.67)	<0.001	0.61	(0.58 to 0.64)	<0.001
Gender	Female	45 607	40.1	1			1		
	Male	53 861	36.5	0.87	(0.84 to 0.89)	<0.001	0.92	(0.89 to 0.95)	<0.001
Ethnic group	White	92 385	38.0	1			1		
	Black	2362	35.7	0.80	(0.73 to 0.87)	<0.001	0.73	(0.65 to 0.82)	<0.001
	S.Asian	2118	44.4	1.24	(1.14 to 1.35)	<0.001	1.04	(0.92 to 1.18)	0.552
	Mixed	458	39.1	0.96	(0.80 to 1.16)	0.677	0.86	(0.71 to 1.04)	0.126
	Other	1641	42.8	1.11	(1.01 to 1.23)	0.039	0.96	(0.85 to 1.09)	0.531
Townsend Deprivation Index, quintiles‡	Q1: Least deprived	19 899	36.2	1			1		
	Q2	19 850	36.9	1.03	(0.99 to 1.07)	0.197	1.02	(0.98 to 1.06)	0.364
	Q3	19 861	36.9	1.02	(0.98 to 1.06)	0.395	1.01	(0.96 to 1.05)	0.783
	Q4	19 868	38.9	1.09	(1.05 to 1.14)	<0.001	1.07	(1.02 to 1.11)	0.003
	Q5: Most deprived	19 869	41.8	1.21	(1.16 to 1.26)	<0.001	1.15	(1.10 to 1.20)	<0.001
Household income, GBP	Greater than 100 000	2529	41.2	1			1		
	52 000 to 1 00 000	11 039	39.6	0.92	(0.85 to 1.01)	0.080	0.91	(0.83 to 1.00)	0.041
	31 000 to 51 999	18 368	37.9	0.89	(0.82 to 0.97)	0.009	0.86	(0.79 to 0.94)	<0.001
	18 000 to 30 999	23 956	37.2	0.91	(0.84 to 0.99)	0.029	0.84	(0.77 to 0.91)	<0.001
	Less than 18 000	26 443	38.6	0.98	(0.90 to 1.07)	0.629	0.82	(0.75 to 0.90)	<0.001
Occupation category§	Professional and administrative	33 464	39.9	1			1		
	Skilled trades	4232	33.9	0.83	(0.77 to 0.88)	<0.001	0.86	(0.80 to 0.92)	<0.001
	Services	5078	40.2	0.97	(0.92 to 1.03)	0.378	0.96	(0.91 to 1.03)	0.263
	Manual and Industrial	6443	35.8	0.88	(0.83 to 0.93)	<0.001	0.88	(0.83 to 0.93)	<0.001
	Other employment	3118	38.3	0.96	(0.89 to 1.03)	0.246	0.99	(0.91 to 1.06)	0.708
	Retired	37 081	35.5	0.93	(0.90 to 0.97)	<0.001	0.93	(0.90 to 0.97)	<0.001
	Unable to work because of sickness or disability	5304	48.5	1.39	(1.31 to 1.48)	<0.001	1.07	(1.00 to 1.14)	0.043
	Unemployed/unanswered	4748	39.8	0.94	(0.88 to 1.00)	0.041	0.91	(0.85 to 0.97)	0.004
Highest level of education (ISCED)¶	5: Tertiary	38 924	38.7	1			1		
	4: Postsecondary non-tertiary	12 224	38.0	0.99	(0.95 to 1.03)	0.648	0.99	(0.95 to 1.04)	0.775
	2–3: Secondary	20 635	38.6	0.98	(0.95 to 1.02)	0.271	0.99	(0.95 to 1.02)	0.438
	1: Primary	25 606	36.9	0.98	(0.95 to 1.01)	0.275	0.94	(0.91 to 0.98)	0.002
Country of birth, by income level**	UK	90 845	37.9	1			1		
	Other high income	2292	41.1	1.11	(1.02 to 1.20)	0.020	1.10	(1.01 to 1.20)	0.038
	Middle income	5108	39.9	1.02	(0.96 to 1.08)	0.470	1.02	(0.93 to 1.12)	0.689
	Low income	1223	42.4	1.11	(0.99 to 1.24)	0.078	1.05	(0.91 to 1.21)	0.512
UK country of residence††	England	88 122	38.5	1			1		
	Scotland	7109	35.4	0.87	(0.83 to 0.91)	<0.001	0.86	(0.82 to 0.91)	<0.001
	Wales	4237	35.4	0.86	(0.80 to 0.92)	<0.001	0.87	(0.82 to 0.93)	<0.001
BMI (categorical)‡‡	Underweight	197	56.9	1.76	(1.32 to 2.34)	<0.001	1.65	(1.24 to 2.20)	<0.001
	Normal (ref)	17 206	41.7	1			1		
	Overweight	41 574	36.9	0.84	(0.81 to 0.87)	<0.001	0.82	(0.79 to 0.85)	<0.001
	Obese	39 887	37.7	0.84	(0.81 to 0.87)	<0.001	0.73	(0.71 to 0.76)	<0.001
Smoking status	Never	48 974	37.7	1			1		
	Previous	40 595	37.5	1.06	(1.03 to 1.09)	<0.001	1.08	(1.05 to 1.11)	<0.001
	Current	9254	43.1	1.26	(1.21 to 1.32)	<0.001	1.24	(1.19 to 1.30)	<0.001
Alcohol units per week (categorical)	None reported	34 563	42.2	1			1		
	Less than 5 units	8024	39.0	0.89	(0.85 to 0.94)	<0.001	0.94	(0.89 to 0.99)	0.014
	5–10 units	14 028	38.8	0.88	(0.85 to 0.92)	<0.001	0.92	(0.88 to 0.96)	<0.001
	10–20 units	19 633	37.2	0.83	(0.80 to 0.86)	<0.001	0.85	(0.82 to 0.89)	<0.001
	20–30 units	10 680	34.2	0.73	(0.69 to 0.76)	<0.001	0.75	(0.71 to 0.78)	<0.001
	More than 30 units	12 540	30.3	0.60	(0.58 to 0.63)	<0.001	0.61	(0.58 to 0.64)	<0.001
Weekly physical activity§§	High (METs >1200)	46 106	37.2	1			1		
	Low (METs ≤1200)	31 134	40.1	1.11	(1.08 to 1.15)	<0.001	1.06	(1.03 to 1.09)	<0.001
No of antihypertensive medications	1	48 501	37.0	1			1		
	2	33 678	39.4	1.14	(1.11 to 1.18)	<0.001	1.15	(1.12 to 1.19)	<0.001
	≥3	13 836	39.8	1.19	(1.14 to 1.24)	<0.001	1.17	(1.13 to 1.22)	<0.001
	Medication list unavailable	3453	35.2	0.93	(0.87 to 1.00)	0.064	0.91	(0.85 to 0.98)	0.014
Family history of CVD	No	34 301	37.1	1			1		
	Yes	65 167	38.7	1.08	(1.05 to 1.11)	<0.001	1.04	(1.01 to 1.07)	0.004
Ever screened for bowel cancer	Yes	38 886	38.0	1			1		
	No	58 407	38.2	0.90	(0.87 to 0.92)	<0.001	0.92	(0.90 to 0.95)	<0.001
No of comorbidities¶¶	0	44 968	31.8	1			1		
	1	36 901	41.3	1.54	(1.50 to 1.58)	<0.001	1.52	(1.48 to 1.57)	<0.001
	2	13 583	46.8	1.95	(1.87 to 2.02)	<0.001	1.89	(1.81 to 1.97)	<0.001
	≥3	4016	50.5	2.21	(2.07 to 2.36)	<0.001	2.09	(1.95 to 2.23)	<0.001

*The number of individuals in levels of a categorical variable may not add up to total n because ‘Do not know’ and ‘Prefer not to answer’ categories have been removed from results table.

†Hypertension control was defined as mean systolic BP ≥140 mm Hg or mean diastolic BP ≥90 mm Hg at baseline assessment, among treated hypertensives.

‡The Townsend index is a measure of material deprivation calculated at the level of census output areas.

§Occupation categories have been condensed from those recorded in UKB. Professional and administrative: managers and senior officials, professional occupations, associate professional and technical occupations, administrative and secretarial occupations. Skilled trades: skilled trades occupations. Services: personal service occupations, sales and customer service occupations. Manual and industrial: process, plant and machine operatives, elementary occupations. Other employment: free text entry that was not coded by UKB.

¶Self-reported highest education achieved was mapped to the ISCED categories.

**Self-reported country of birth was mapped to the World Bank Analytical Classifications for calendar year 2010.

††The assessment centre the participant attended was used as a proxy for country of residence.

‡‡BMI has been categorised as: underweight <18.5 kg/m^2^; normal 18.5–24.9 kg/m^2^; overweight 25.0–29.9 kg/m^2^; obese ≥30.0 kg/m^2^.

§§The total MET minutes per week is based on self-reported frequency and duration of walking, moderate and vigorous activity, and was then dichotomised based on WHO physical activity guideline thresholds.

¶¶The conditions of interest that counted towards number of comorbidities were: CVD, diabetes, arrhythmia (afib/flutter), asthma or COPD, migraines, epilepsy, anxiety, depression, osteoarthritis, other joint disorder.

BMI, body mass index; BP, blood pressure; COPD, chronic obstructive pulmonary disease; CVD, cardiovascular disease; ISCED, International Standard Classification of Education; MET, metabolic equivalent task; UKB, UK Biobank.

Hypertension control was also inversely associated with characteristics that reflect lower socioeconomic standing, including: low income (OR 0.82, 95% CI 0.75 to 0.90 for those with an annual household income of £18 000 compared with a reference group of >£100 000), low education (OR 0.94, 95% CI 0.91 to 0.98 for those educated to primary school level only compared with those who reached tertiary level), and less professionalised occupations (OR 0.88, 95% CI 0.83 to 0.93 for manual and industrial occupations compared with professional and senior administrative occupations). Paradoxically, however, individuals who lived in the most materially deprived areas (based on the Townsend index) and those with lower physical activity had higher odds of hypertension control. Residence in Scotland (OR 0.86, 95% CI 0.82 to 0.91) or Wales (OR 0.87, 95% CI 0.82 to 0.93) was associated with slightly lower odds of hypertension control compared with residence in England.

Smoking, a CVD risk factor, was associated with higher odds of hypertension control (OR 1.24, 95% CI 1.19 to 1.30 for current smokers compared with those who had never smoked). The characteristic most strongly associated with hypertension control was having ≥3 comorbidities (OR 2.09, 95% CI 1.95 to 2.23; table 2). When considering the individual comorbidities (table 3), those most strongly associated with hypertension control were known CVD (OR 2.11, 95% CI 2.04 to 2.19), atrial fibrillation or atrial flutter (OR 1.72, 95% CI 1.56 to 1.90), migraines (OR 1.68, 95% CI 1.56 to 1.81), anxiety (OR 1.47, 95% CI 1.34 to 1.62), diabetes (OR 1.32, 95% CI 1.27 to 1.36) and depression (OR 1.27, 95% CI 1.20 to 1.34).

**Table 3:** Multivariable logistic regression examining the association of individual comorbidities with hypertension control, among middle-aged UK adults on anti-hypertensive treatment (N=99 468)

				Age and sex adjusted			Multivariable adjusted*		
Coefficient	Level	n	% controlled†	OR	95% CI	P value	OR	95% CI	P value
CVD	No	80 124	34.9	1			1		
	Yes	19 344	51.3	2.20	(2.12 to 2.27)	<0.001	2.11	(2.04 to 2.19)	<0.001
Diabetes	No	84 040	37.1	1			1		
	Yes	15 428	43.9	1.36	(1.31 to 1.41)	<0.001	1.32	(1.27 to 1.36)	<0.001
Arrhythmia (afib/flutter)	No	97 769	37.9	1			1		
	Yes	1699	49.0	1.70	(1.55 to 1.87)	<0.001	1.72	(1.56 to 1.90)	<0.001
Asthma or COPD	No	86 889	38.0	1			1		
	Yes	12 579	38.8	1.01	(0.97 to 1.05)	0.650	0.96	(0.92 to 1.00)	0.031
Migraines	No	96 500	37.7	1			1		
	Yes	2968	52.7	1.68	(1.56 to 1.81)	<0.001	1.68	(1.56 to 1.81)	<0.001
Epilepsy	No	98 631	38.1	1			1		
	Yes	837	41.3	1.10	(0.96 to 1.26)	0.178	0.96	(0.83 to 1.10)	0.554
Anxiety	No	97 564	37.9	1			1		
	Yes	1904	49.7	1.51	(1.38 to 1.66)	<0.001	1.47	(1.34 to 1.62)	<0.001
Depression	No	93 353	37.5	1			1		
	Yes	6115	47.2	1.40	(1.33 to 1.48)	<0.001	1.27	(1.20 to 1.34)	<0.001
Osteoarthritis	No	87 749	37.9	1			1		
	Yes	11 719	39.7	1.10	(1.06 to 1.15)	<0.001	1.08	(1.04 to 1.13)	<0.001
Other joint disorder	No	95 070	38.1	1			1		
	Yes	4398	38.6	1.02	(0.96 to 1.08)	0.597	0.97	(0.91 to 1.03)	0.313

*The multiply-adjusted model presented in table 3 contains variables included in table 2 except for the number of comorbidities, and additionally contains the individual types of comorbidities.

†Thus, variables included in the model above are: age group, years, gender, ethnic group, Townsend Deprivation Index, quintiles, household Income, GBP, occupation category, highest level of education (ISCED), country of birth, by income level, UK country of residence, BMI (categorical), smoking status, alcohol units per week (categorical), weekly physical activity, number of antihypertensive medications, family history of CVD, diabetes, arrhythmia (afib/flutter), asthma or COPD, migraines, epilepsy, anxiety, depression, osteoarthritis, other joint disorder, ever screened for bowel cancer. Afib/flutter: atrial fibrillation or atrial flutter.

BMI, body mass index; COPD, chronic obstructive pulmonary disease; CVD, cardiovascular disease; ISCED, International Standard Classification of Education.

Results from analyses using the second BP measurement alone, stratified by 10-year age groups (given that the majority of UKB participants were 60–69 years old), stratified by the number of comorbidities and CVD status (online supplemental table 3), and including UKB participants with serious comorbidities did not alter conclusions drawn from the main analysis.

## Discussion

### Principal findings

Our analysis found that only two out of five treated middle-aged hypertensives were controlled in a high income country (HIC) setting, and revealed that hypertension risk factors and characteristics of lower socioeconomic status were inversely associated hypertension control. We also found that having comorbidities was positively associated with hypertension control, including comorbidities not linked with increased CVD risk.

### Strengths and weaknesses of the study

This is one of the largest population-based analyses of hypertension control in middle-aged adults, and has used comprehensive sociodemographic and medical history data available from the UKB to investigate topics of emerging public health priority (performance of hypertension control in the ageing population and the role of comorbidities). That said, our study has several notable limitations. First, we relied on self-reported information on comorbidities and medications. This may have resulted in differential misclassification, even if mitigated by the in-person interview and review of a medication list by a nurse. Second, UKB baseline data are now a decade old and do not directly include information on factors known to be associated with hypertension control such as medication adherence and healthcare utilisation. Third, participation in the UKB was by volunteers for a longitudinal study and required visiting study assessment centres. Studies have reported evidence of healthy volunteer selection bias and limitations in national representativeness of the UKB study population thus our prevalence estimates should be interpreted with this in mind. More specifically, UKB participants have been found to differ from UK nationally representative surveys with respect to several socioeconomic (eg, more liked to be educated), lifestyle and clinical characteristics (eg, less likely to be obese).^4^ Our estimated prevalence of hypertension control may thus not accurately reflect prevalence in the UK’s general population aged 40–69 years, and might be anticipated to overestimate this prevalence. While not nationally representative, UKB is a population-based study with a large number of participants who have heterogeneous exposure levels that are assessed with high internal validity. As such, UKB is a suitable resource for providing valid inferences of exposure–outcome associations that are generalisable to other populations,^4^ and for our research objective to identify factors associated with hypertension control.

### Prevalence: comparison with other studies and meaning of study findings

Comparisons between studies of hypertension control prevalence rates are limited by differing age groups reported, noting that hypertension control tends to be lower among older adults compared with younger adults treated for hypertension. Nonetheless, our estimate of hypertension control is comparable with those reported in multicountry studies during UKB’s recruitment period: a 40.7% hypertension control (defined similarly) average among HICs adults aged 35–70 years enrolled 2003–2009 was reported in the PURE (Prospective Urban Rural Epidemiology) study^5^; a systemic review of population-based studies from 90 countries reported hypertension control proportions of 38.6% (95% CI 25.5% to 51.6%) in 2000 and 50.4% (95% CI 44.4% to 56.4%) in 2010 for HIC adults aged 18+years in.^23^ Our estimates are lower than those from Health Survey England (HSE), which in 2008 reported hypertension control prevalence of 58.7% for ages 45–54 years and 57.5% for ages 65–74 years. HSE reports also indicate an improvement over time in hypertension control prevalence, with 2018 estimates of 63.9% for ages 45–54 years for ages 55–64 years, 54.6% and 67.7% for ages 65–74 years. This discrepancy with our estimates may be due to a combination of differences in age-group cut-offs for estimates reported, differences in how hypertension was defined (HSE did not include self-reported hypertension), and that UKB is not nationally representative.^14^ This notwithstanding, a recent *Lancet* publication describing trends in hypertension control (employing nationally representative surveys) in 12 HICs found that improvement in hypertension control rates over time has plateaud and that UK’s hypertension control performance was poorer than for the US, Germany and Canada.^24^ These figures highlight the continued gaps and continued need for identifying factors associated with control (or lack thereof) that might inform healthcare services improvement efforts.

The nature of these efforts would need to be informed by updated analyses that include operational data on the design of healthcare delivery, process measures for compliance with clinical guidelines and patient adherence with prescribed medicines. While we applied the BP target of 140/90 mm Hg consistent with clinical guidelines in practice during UKB’s recruitment period (NICE 2006), studies have now demonstrated CVD benefit of reducing systolic BP below 120 mm Hg.^25^ That said, we recognise that the 140/90 mm Hg threshold used did not distinguish between types of hypertension such as isolated systolic hypertension, and was lower than the practice guidance linked to pay-for-performance reimbursement in 2006. The UK’s General Medical Services 2006/2007 contract contained the indicator: ‘The percentage of patients with hypertension in whom the last BP (measured in the previous 9 months) is ≤150/90 mm Hg’.^26^ It is for this reason that we included additional analysis using the less stringent <160/100 mm Hg threshold for control. With this threshold, a fifth of treated hypertensives were uncontrolled and the associations observed were similar to those in our primary analysis with the exception of gender (for which there was no statistically significant difference in control between men and women).

### Associations: comparison with other studies and meaning of study findings

The relationships we observed between hypertension control and hypertension risk factors such as age, obesity, black ethnicity and alcohol use are consistent with results from previous studies in the UK^7 27^ and in other HICs.^8 28 29^ Contributors to poorer control among older adults include increasing vascular stiffness with age, the possible reluctance of providers to intensify medications, and barriers associated with polypharmacy.^8^ Reasons for poorer control in Black people include higher prevalence of resistant hypertension^30^ and structural factors perhaps not well captured by the variables included in our models (eg, neighbourhood deprivation). Lower socioeconomic status has also been linked with poorer hypertension control,^31^ possibly due to a combination of less access to care (eg, less flexible employment situation, longer distance to travel), differential treatment and/or poorer quality of services, lower health literacy, and more chronic stress. Differences in the odds of hypertension control between England, Scotland and Wales are likely due to regional differences in lifestyle behaviour or healthcare utilisation patterns not accounted for.

To explore the possibility of collider bias introduced by conditioning our analysis on treatment, we performed multivariable logistic regression for the same correlates for hypertension control among all hypertensives (treated and untreated). Results of this analysis were similar to our main analysis results (among treated hypertensives), thus did not support a collider effect by treatment, but we cannot rule this out as a function of selection into UKB.

With regards to comorbidities, our findings are consistent other studies.^7 27^ In a cross-sectional analysis of UK primary care data of 31 676 adults in a single London borough diagnosed with hypertension, Sarkar *et al* found that the number of comorbidities was the strongest predictor of systolic BP and that systolic BP was lower with multimorbidity regardless of the type of comorbidity (including diabetes).^7^ Our analysis expands on these findings by covering a larger geographic area and including a larger sample size and additional explanatory variables.

Hypertensives with comorbidities linked to CVD risk may be better controlled due to providers appropriately managing them more aggressively.^8^ This hypothesis is supported by our finding that smokers and those have migraines are more likely to be controlled, and that a higher percentage of participants with CVD risk factors were on ≥3 antihypertensives (online supplemental table 1). There are several possible explanations for the association between hypertension control and comorbidities that are not linked to CVD with respect to pathophysiology, risk factor profile, or management pathway (ie, discordant comorbidities). A leading explanation is the confounding effect of frequent healthcare utilisation, which has been associated with higher hypertension control.^8^ This might happen through exposure to health promotion and medication adherence counselling by providers. It could also be through opportunistic BP screening such that those with comorbidities are diagnosed at an earlier stage of hypertension (with corresponding lower BP).

### Unanswered questions and future research

More studies are needed to investigate the mechanisms underlying the associations between hypertension control and discordant comorbidities, as well as to understand challenges faced by groups with lower odds of hypertension control. Better performance with hypertension control would be expected to reduce CVD-related deaths, but could also potentially lower mortality in the ongoing COVID-19 pandemic, given that hypertension has been linked with poorer outcomes in the setting of COVID-19 infection.^32^

## Conclusions

In one of the largest population-based analyses in middle-aged adults, the majority of individuals treated for hypertension were not controlled. Older, black and lower-income hypertensives were less likely to be controlled, while those with multimorbidity and at increased CVD risk were more likely to be controlled. More research is needed to understand barriers to hypertension control, and the mechanisms underlying the association between hypertension control and comorbidities not linked with increased CVD risk.
