# Supplementary material for: Prevalence and determinants of hypertension control among almost 100 000 treated adults in the UK
Source: Open Heart. 2021 Mar 11;8(1):e001461. doi: 10.1136/openhrt-2020-001461 (PMC7957140; doi:10.1136/openhrt-2020-001461)
Supplement: Supplementary data [file openhrt-2020-001461supp001.pdf]

SUPPLEMENTARY TABLES

Supplementary Table 1. Distribution of the number of antihypertensives by CVD risk level (n 99,468)

	CVD	Higher risk	Lower risk	Sum
1	7,078 (36.59%)	463 (42.01%)	40,960 (51.83%)	48,501
2	7,878 (40.73%)	379 (34.39%)	25,421 (32.17%)	33,678
>=3	3,740 (19.33%)	210 (19.06%)	9,886 (12.51%)	13,836
0 (No antihypertensives reported)	640 (3.31%)	49 (4.45%)	2,395 (3.03%)	3,084
NA (No medications reported)	8 (0.04%)	1 (0.09%)	360 (0.46%)	369
Sum	19,344	1,102	79,022	99,468

Individuals classified as higher risk were those without prior diagnosis of CVD (self-report), and were aged ≥60 years, and were obese (BMI >30), and were current smokers.

Tapela N, *et al. Open Heart* 2021; 8:e001461. doi: 10.1136/openhrt-2020-001461

Supplementary Table 2. Concordance between BP measurements at baseline and follow up within three years, using BP threshold of 140/90 mmHg (n 2,134).

	Measured BP < 140/90 at repeat visit	Measured BP >= 140/90 at repeat visit	Sum
Measured BP < 140/90 at baseline	892 (79.79%)	226 (20.21%)	1,118
Measured BP >= 140/90 at baseline	273 (26.87%)	743 (73.13%)	1,016
Sum	1,165	969	2,134

The Cohen's kappa here is 0.5302715 which indicates "moderate agreement".
The Spearman correlation coefficient is 0.5307913.

Supplementary Table 3. Multivariable logistic regression identifying factors associated with hypertension control stratified by number of comorbidities and prior CVD status, among middle-aged UK adults on anti-hypertensive treatment

	No comorbidities					2+ comorbidities without CVD					2+ comorbidities with CVD				
Coefficient	n	% controlled	OR	95% CI	p	n	% controlled	OR	95% CI	p	n	% controlled	OR	95% CI	p
Age group, years															
40-49	3750	38.0	1			775	55.5	1			381	64.8	1		
50-59	13166	35.7	0.89	(0.82, 0.96)	0.002	2586	47.7	0.77	(0.66, 0.91)	0.002	2138	56.9	0.73	(0.58, 0.92)	0.008
60-69	28052	29.1	0.66	(0.61, 0.72)	<0.001	4991	39.1	0.57	(0.48, 0.68)	<0.001	6728	49.2	0.58	(0.46, 0.73)	<0.001
Gender															
Female	21460	35.3	1			5032	45.6	1			3047	52.3	1		
Male	23508	28.6	0.78	(0.75, 0.82)	<0.001	3320	39.7	0.85	(0.77, 0.95)	0.002	6200	51.3	1.04	(0.95, 1.15)	0.392
Ethnic group															
White	41829	31.7	1			7728	42.8	1			8560	51.4	1		
Black	1230	32.0	0.74	(0.62, 0.87)	<0.001	186	45.7	1.01	(0.67, 1.50)	0.980	134	49.3	0.75	(0.48, 1.19)	0.224
S. Asian	767	35.7	1.00	(0.82, 1.23)	0.987	177	55.9	1.64	(1.07, 2.52)	0.025	318	55.7	1.03	(0.68, 1.55)	0.895
Mixed	192	26.0	0.61	(0.44, 0.86)	0.004	45	53.3	1.18	(0.64, 2.16)	0.594	42	57.1	1.13	(0.60, 2.13)	0.699
Other	735	37.0	0.99	(0.82, 1.20)	0.930	159	48.4	1.09	(0.72, 1.65)	0.681	142	55.6	0.94	(0.60, 1.47)	0.790
Townsend Deprivation Index, quintiles[3]															
Q1: Least deprived	9985	30.7	1			1381	41.8	1			1372	51.5	1		
Q2	9546	30.8	1.01	(0.95, 1.07)	0.859	1467	40.4	0.94	(0.81, 1.09)	0.427	1540	52.9	1.05	(0.91, 1.22)	0.480
Q3	9243	31.0	1.02	(0.95, 1.08)	0.613	1580	41.5	0.96	(0.83, 1.12)	0.629	1620	49.3	0.92	(0.80, 1.07)	0.275
Q4	8729	32.8	1.09	(1.03, 1.17)	0.005	1761	44.3	1.07	(0.92, 1.24)	0.369	1947	49.9	0.93	(0.80, 1.07)	0.304
Q5: Most deprived	7411	34.5	1.20	(1.12, 1.29)	<0.001	2154	46.6	1.14	(0.98, 1.33)	0.081	2757	53.6	1.05	(0.91, 1.21)	0.479
Household Income, GBP															
Greater than 100,000	1456	36.7	1			130	50.0	1			99	56.6	1		
52,000 to 100,000	6005	34.3	0.89	(0.79, 1.01)	0.065	634	45.9	0.80	(0.55, 1.18)	0.265	523	56.2	0.99	(0.63, 1.53)	0.950
31,000 to 51,999	9323	32.0	0.83	(0.74, 0.93)	0.002	1246	45.7	0.80	(0.55, 1.15)	0.226	1142	51.8	0.89	(0.58, 1.36)	0.584
18,000 to 30,999	11054	30.8	0.81	(0.72, 0.92)	<0.001	1906	41.3	0.69	(0.48, 1.00)	0.050	2047	51.7	0.94	(0.62, 1.43)	0.767
Less than 18,000	9753	30.4	0.79	(0.69, 0.89)	<0.001	2818	43.6	0.71	(0.49, 1.03)	0.071	3659	51.1	0.90	(0.59, 1.36)	0.605
Occupation category[4]															
Professional and Administrative	17182	34.6	1			2375	45.5	1			1912	54.7	1		
Skilled trades	2084	27.6	0.87	(0.78, 0.97)	0.011	237	35.9	0.85	(0.64, 1.13)	0.265	326	46.6	0.76	(0.60, 0.97)	0.028
Services	2358	34.2	0.93	(0.85, 1.02)	0.133	503	45.1	0.96	(0.78, 1.17)	0.661	316	57.6	1.17	(0.91, 1.50)	0.212
Manual and Industrial	3052	28.7	0.86	(0.78, 0.94)	<0.001	457	46.2	1.16	(0.94, 1.44)	0.169	579	51.1	0.91	(0.75, 1.11)	0.371
Other employment	1550	34.0	1.03	(0.92, 1.15)	0.582	219	38.4	0.82	(0.61, 1.10)	0.180	185	56.8	1.14	(0.84, 1.56)	0.397
Retired	15838	28.9	0.91	(0.87, 0.97)	0.002	3026	39.5	1.00	(0.88, 1.13)	0.947	4077	48.6	0.89	(0.79, 1.01)	0.066
Unable to work	796	39.3	1.24	(1.06,	0.006	1050	49.6	1.13	(0.95,	0.164	1495	54.8	0.96	(0.82,	0.595

Tapela N, *et al. Open Heart* 2021; 8:e001461. doi: 10.1136/openhrt-2020-001461

because of sickness or disability				1.44)					1.34)					1.12)	
Unemployed/unanswered	2108	32.8	0.88	(0.80, 0.98)	0.016	485	43.9	0.86	(0.70, 1.06)	0.156	357	54.6	0.94	(0.74, 1.19)	0.601
Highest level of education (ISCED)[5]															
5: Tertiary	18640	32.9	1			3043	44.6	1			2944	53.4	1		
4: Post-secondary non-tertiary	5672	32.9	1.01	(0.95, 1.08)	0.716	1041	42.7	0.96	(0.83, 1.11)	0.612	1002	48.5	0.86	(0.74, 1.00)	0.044
2-3: Secondary	9793	32.1	0.94	(0.89, 1.00)	0.040	1704	45.2	1.00	(0.88, 1.13)	0.959	1715	54.9	1.06	(0.94, 1.20)	0.337
1: Primary	9917	29.1	0.90	(0.85, 0.96)	<0.001	2395	40.6	0.88	(0.77, 0.99)	0.040	3379	49.6	0.89	(0.80, 1.00)	0.042
Country of birth, by income level[6]															
UK	40966	31.5	1			7638	42.8	1			8466	51.4	1		
Other high income	1177	36.3	1.10	(0.98, 1.25)	0.118	181	49.7	1.18	(0.87, 1.61)	0.283	161	57.1	1.18	(0.85, 1.64)	0.311
Middle income	2305	33.9	1.04	(0.90, 1.20)	0.611	424	48.1	0.90	(0.64, 1.25)	0.521	491	54.4	1.02	(0.71, 1.47)	0.899
Low income	520	34.2	1.02	(0.82, 1.28)	0.845	109	47.7	0.87	(0.54, 1.40)	0.554	129	51.9	0.88	(0.54, 1.44)	0.617
UK country of residence[7]															
England	39774	32.2	1			7515	43.6	1			8103	52.0	1		
Scotland	3242	28.0	0.80	(0.74, 0.87)	<0.001	478	40.6	0.86	(0.71, 1.04)	0.124	746	49.3	0.88	(0.75, 1.03)	0.102
Wales	1952	30.7	0.94	(0.85, 1.03)	0.196	359	40.7	0.90	(0.72, 1.12)	0.324	398	48.7	0.88	(0.72, 1.09)	0.238
BMI (categorical)[8]															
Underweight	96	50.0	1.58	(1.05, 2.37)	0.027	18	55.6	1.35	(0.52, 3.50)	0.533	14	78.6	2.39	(0.65, 8.70)	0.188
Normal (ref)	9183	35.5	1			970	47.9	1			1139	57.1	1		
Overweight	20358	30.4	0.82	(0.78, 0.87)	<0.001	2597	43.3	0.91	(0.78, 1.06)	0.214	3380	52.5	0.84	(0.74, 0.97)	0.016
Obese	15140	31.3	0.80	(0.75, 0.84)	<0.001	4694	42.3	0.80	(0.69, 0.93)	0.003	4615	49.6	0.72	(0.63, 0.83)	<0.001
Smoking status															
Never	23984	32.1	1			4091	44.2	1			3276	51.8	1		
Previous	17040	30.5	1.06	(1.01, 1.11)	0.013	3358	41.6	1.05	(0.95, 1.15)	0.369	4606	49.9	1.00	(0.91, 1.10)	0.988
Current	3709	35.3	1.26	(1.17, 1.36)	<0.001	830	46.5	1.09	(0.93, 1.28)	0.283	1279	56.9	1.22	(1.06, 1.40)	0.005
Alcohol units per week (categorical)															
None reported	13016	34.7	1			4037	47.0	1			4084	53.7	1		
Less than 5 units	3898	34.2	0.98	(0.91, 1.06)	0.629	642	42.1	0.84	(0.70, 1.00)	0.044	567	52.9	0.99	(0.82, 1.18)	0.873
5 to 10 units	6896	33.3	0.94	(0.88, 1.00)	0.068	1010	44.4	0.93	(0.80, 1.07)	0.302	1053	52.7	0.96	(0.83, 1.10)	0.540
10 to 20 units	9711	31.5	0.88	(0.83, 0.94)	<0.001	1223	40.3	0.79	(0.68, 0.90)	<0.001	1617	51.5	0.90	(0.79, 1.01)	0.082
20 to 30 units	5344	28.9	0.79	(0.73, 0.85)	<0.001	655	36.8	0.69	(0.58, 0.83)	<0.001	892	48.9	0.80	(0.69, 0.94)	0.005
More than 30 units	6103	25.5	0.66	(0.62, 0.72)	<0.001	785	33.9	0.58	(0.49, 0.69)	<0.001	1034	44.4	0.65	(0.56, 0.76)	<0.001
Weekly physical activity[9]															
High (METs > 1200)	22166	31.3	1			3209	42.6	1			3637	50.6	1		
Low (METs <= 1200)	13273	33.6	1.07	(1.02, 1.12)	0.008	2994	44.1	1.04	(0.94, 1.16)	0.439	3332	53.1	1.10	(1.00, 1.21)	0.056
Number of antihypertensive medications															
1	23417	30.5	1			4254	45.7	1			3254	51.3	1		
2	14850	33.0	1.19	(1.14, 1.25)	<0.001	2543	40.0	0.84	(0.76, 0.93)	<0.001	3617	54.5	1.18	(1.07, 1.30)	<0.001

>=3	5183	35.1	1.38	(1.30, 1.48)	<0.001	1235	41.9	0.95	(0.83, 1.08)	0.448	2055	47.6	0.92	(0.82, 1.03)	0.137
Medication list unavailable	1518	29.5	0.98	(0.87, 1.10)	0.731	320	42.8	0.88	(0.69, 1.11)	0.278	321	48.9	0.89	(0.70, 1.12)	0.312
Family history of CVD															
No	16531	31.1	1			2840	44.0	1			2383	50.6	1		
Yes	28437	32.2	1.03	(0.99, 1.08)	0.133	5512	42.9	0.98	(0.90, 1.08)	0.743	6864	52.0	1.06	(0.97, 1.17)	0.208
Ever screened for bowel cancer															
Yes	17055	31.5	1			3436	42.6	1			4003	49.9	1		
No	27061	32.0	0.91	(0.87, 0.95)	<0.001	4708	44.0	0.93	(0.84, 1.02)	0.127	4976	52.8	1.06	(0.97, 1.15)	0.210

Note: *[1] Number of individuals in levels of a categorical variable may not add up to total n because 'Do not know' and 'Prefer not to answer' categories have been removed from results table [2] Hypertension control is defined as mean systolic BP >= 140 mmHg or mean diastolic BP >= 90 mmHg at baseline assessment, among treated hypertensives. [3] The Townsend index is a measure of material deprivation calculated at the level of census output areas. [4] Occupation categories have been condensed from those recorded in UKB. Professional and Administrative: Managers and Senior Officials, Professional Occupations, Associate Professional and Technical Occupations, Administrative and Secretarial Occupations. Skilled trades: Skilled Trades Occupations. Services: Personal Service Occupations, Sales and Customer Service Occupations. Manual and Industrial: Process, Plant and Machine Operatives, Elementary Occupations. Other employment: free text entry that was not coded by UKB. [5] Self-reported highest education achieved was mapped to the International Standard Classification of Education (ISCED) categories [6] Self-reported country of birth was mapped to the World Bank Analytical Classifications for calendar year 2010. [7] The assessment centre the participant attended was used as a proxy for country of residence [8] BMI has been categorised as: Underweight < 18.5 kg/m^2; Normal 18.5 - 24.9 kg/m^2; Overweight 25.0 - 29.9 kg/m^2; Obese >= 30.0 kg/m^2. [9] The total Metabolic Equivalent Task (MET) minutes per week is based on self-reported frequency and duration of walking, moderate and vigorous activity, and was then dichotomized based on WHO physical activity guideline thresholds. [10] The number of comorbidities considered to be of interest - cardiovascular disease, diabetes, arrhythmia (afib/flutter), asthma or COPD, migraines, epilepsy, anxiety, depression, osteoarthritis, other joint disorder.*

Tapela N, *et al. Open Heart* 2021; 8:e001461. doi: 10.1136/openhrt-2020-001461
