# Supplementary material for: Prevalence and determinants of hypertension control among almost 100 000 treated adults in the UK
Source: Open Heart. 2021 Mar 11;8(1):e001461. doi: 10.1136/openhrt-2020-001461 (PMC7957140; doi:10.1136/openhrt-2020-001461)
Supplement: Supplementary data [file openhrt-2020-001461supp002.pdf]

Supplementary Figure 1. Flowchart illustrating selection of analytic dataset, using BP threshold of BP 160/100 to define hypertension diagnosis and control (n 99,468)

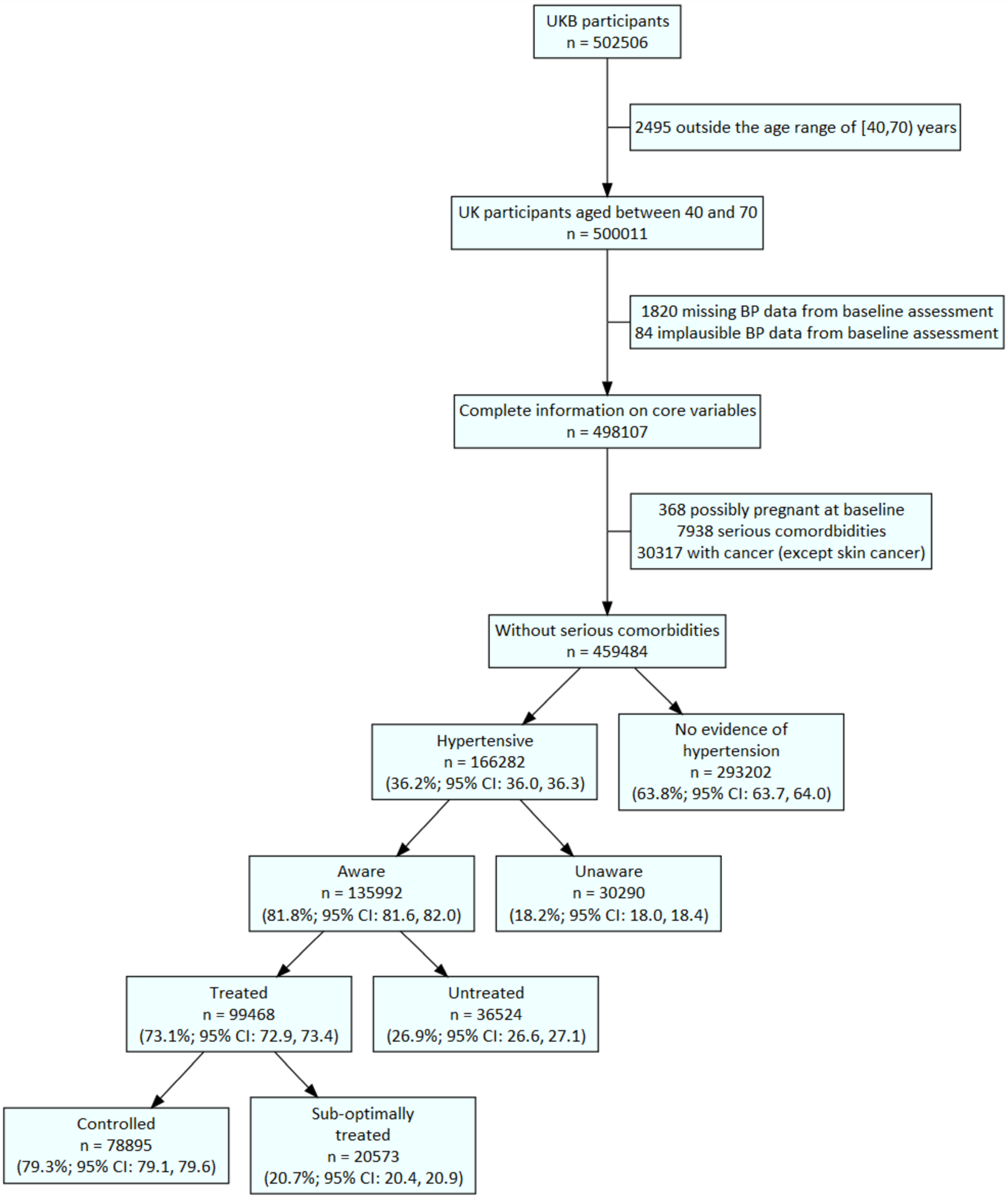

Missing BP data comprises: having fewer than 2 BP measurements at baseline assessment, or missing responses to questions on hypertension history. The following BP values were deemed implausible (defined as previously reported(15): systolic BP < 70 mmHg or ≥ 270 mmHg, diastolic BP < 50 mmHg or ≥ 150 mmHg. Hypertension was defined as self-report of hypertensive medication use, or self-report of a prior diagnosis of hypertension, or mean BP ≥160/100 mmHg at baseline assessment. Awareness was defined as report of a prior diagnosis of hypertension by a health professional, among those who were hypertensive. Treatment was defined as report of hypertensive medication among those who were aware. Control was defined as mean BP < 160/100 mmHg at baseline assessment, among those who were treated.

Tapela N, *et al. Open Heart* 2021; 8:e001461. doi: 10.1136/openhrt-2020-001461
