# Supplementary material for: Prevalence and determinants of hypertension control among almost 100 000 treated adults in the UK
Source: Open Heart. 2021 Mar 11;8(1):e001461. doi: 10.1136/openhrt-2020-001461 (PMC7957140; doi:10.1136/openhrt-2020-001461)
Supplement: Supplementary data [file openhrt-2020-001461supp003.pdf]

Supplementary Figure 2. Scatterplot of blood pressure among treated hypertensives (n 99,468), categorized according to BP threshold 140/90 mmHg.

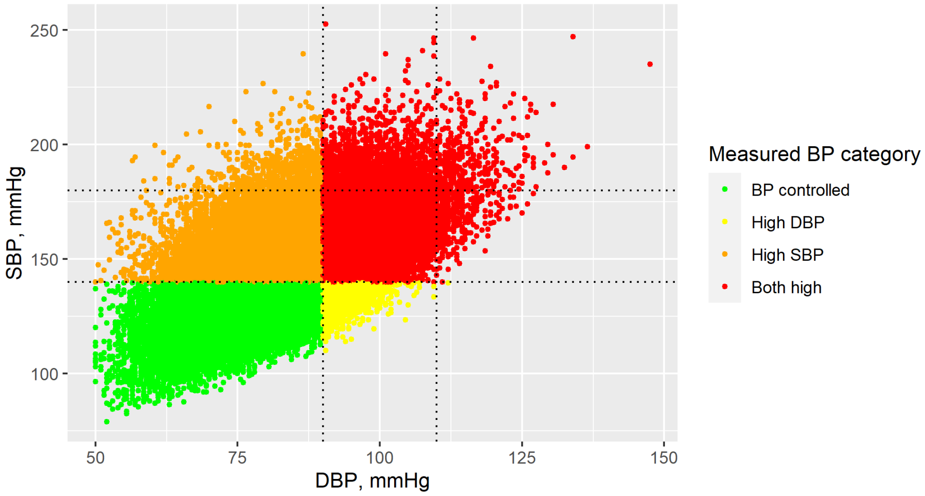

Among diagnosed and treated hypertensives, 399 (0.40%) had both SBP ≥ 180 and DBP ≥ 110 ; 3754 (3.77%) had SBP ≥ 180 or DBP ≥110; 3367 (3.39%) had SBP ≥ 180 only; 786 (0.79%) had DBP ≥ 110 only.

Tapela N, *et al. Open Heart* 2021; 8:e001461. doi: 10.1136/openhrt-2020-001461
